# A redox-related lncRNA signature in bladder cancer

**DOI:** 10.1038/s41598-024-80026-9

**Published:** 2024-11-16

**Authors:** Fuguang Zhao, Hui Xie, Yawei Guan, Jingfei Teng, Zhihui Li, Feng Gao, Xiao Luo, Chong Ma, Xing Ai

**Affiliations:** 1 Department of Urology, The Third Medical Center, Chinese People’s Liberation Army (PLA) General Hospital, Beijing, 100039 P.R. China; 2 Department of Urology, The Seventh Medical Center, Chinese People’s Liberation Army (PLA) General Hospital, Beijing, 100700 P.R. China; 3 Department of Urology, The First Affiliated Hospital of Fujian Medical University, Fuzhou, 350005 P.R. China

**Keywords:** Redox, lncRNA, Bladder cancer, Chemotherapy, Bladder cancer, Bioinformatics

## Abstract

The redox status is intricately linked to the development and progression of cancer, a process that can be modulated by long non-coding RNAs (lncRNAs). Previous studies have demonstrated that redox regulation can be considered a potential therapeutic approach for cancer. However, the redox-related lncRNA predictive signature specific to bladder cancer (BCa) has yet to be fully elucidated. The purpose of our study is to establish a redox-related lncRNA signature to improve the prognostic prediction for BCa patients. To achieve this, we downloaded transcriptome and clinical data from the Cancer Genome Atlas (TCGA) database. Prognostic redox-related lncRNAs were identified through univariate Cox regression, least absolute shrinkage and selection operator (LASSO) regression, and multivariate Cox regression analysis, resulting in the establishment of two risk groups. A comprehensive analysis corresponding to clinical features between high-risk and low-risk groups was conducted. Eight redox-related lncRNAs (AC018653.3, AC090229.1, AL357033.4, AL662844.4, AP003352.1, LINC00649, LINC01138, and MAFG-DT) were selected to construct the risk model. The overall survival (OS) in the high-risk group was worse than that in the low-risk group (*p* < 0.001). The redox-related lncRNA signature exhibits superior predictive accuracy compared to traditional clinicopathological characteristics. Gene Set Enrichment Analysis (GSEA) showed that the MAPK signaling pathway and Wnt signaling pathway were enriched in the high-risk group. Compared with the low-risk group, patients in the high-risk group demonstrated increased sensitivity to cisplatin, docetaxel, and paclitaxel. Furthermore, IGF2BP2, a potential target gene of MAFG-DT, was found to be overexpressed in tumor tissues and correlated with overall survival (OS). Our study demonstrated that the predictive signature based on eight redox-related lncRNAs can independently and accurately predict the prognosis of BCa patients.

**Supplementary Information:**

The online version contains supplementary material available at 10.1038/s41598-024-80026-9.

## Introduction

Bladder cancer (BCa) is the second most common urological malignancy and the 10th most common cancer worldwide, with more than 550,000 newly diagnosed cases every year^1,2^. Current therapeutic options encompass surgery, chemotherapy, radiotherapy, and immunotherapy, based on the histological categories and disease stages. Approximately 50% of BCa patients will experience recurrence or develop metastases after surgical intervention^3–5^, and only a subset of patients responds effectively to chemotherapy and immunotherapy^6,7^. Currently, there is a paucity of reliable biomarkers that can be used for prognostic prediction, which complicates the identification of patients who would benefit most from specific treatments. Hence, the development of a new prognostic signature to improve prognostic prediction and individual-based therapy for BCa is required.

A growing number of studies have revealed the involvement of redox homeostasis in various biological processes, highlighting its importance for physical health^8^. Disrupting this homeostasis, particularly through the accumulation of reactive oxygen species (ROS), often induces oxidative stress, contributing to malignant behavior in various cancer types^9,10^. In fact, ROS have been demonstrated to be integral to tumor progression and survival^11,12^. Nevertheless, the role of ROS in cancer is multifaceted and concentration-dependent. Moderate ROS levels promote cancer cell survival and progression, whereas excessive ROS levels are cytotoxic to cancer cells^13,14^. Therefore, modulating ROS levels might be a pivotal approach for cancer treatment. In the BCa microenvironment, elevated ROS levels, to which tumor cells are particularly susceptible, may result in cell death through ROS-initiated cell death signaling pathways or via the direct damaging effects of ROS^15–19^. Hence, focusing on ROS overexpression, particularly through the use of pro-oxidants, could represent a promising treatment strategy for BCa^20^.

The long non-coding RNAs (lncRNAs) refers to a kind of non-coding RNAs with more than 200 nucleotides in length^21^. Despite lacking protein-coding capacity, lncRNAs participate in almost the whole life cycle of cells through various cellular processes, including the cell cycle, cell differentiation, cellular signal transduction, metabolism, and other related processes^22,23^. Additionally, many lncRNAs are crucial for cell differentiation and development in animals, as demonstrated primarily through their involvement in the control of chromatin architecture, the enhancer action, and the formation of biomolecular condensates^24^. Considering lncRNAs perform a wide range of functions, their dysregulation is linked to a variety of human diseases, including neurological diseases and cancer^23,25^. Therefore, studying the associations between lncRNAs and complex diseases is crucial for improving the diagnosis, treatment, prognosis, and prevention of lncRNA-related diseases. Computational methods can be applied to the study of lncRNA-disease or microRNA-disease associations^23,26–29^. Compared to traditional experimental methods, computational approaches reduce time and cost while greatly improving the efficiency of research^23,30,31^.

In BCa, lncRNAs play a vital role in determining cancer stage, grade, progression, and overall patient survival^32–34^. Notably, recent studies have shown that lncRNAs are closely associated with oxidative stress alteration, which inhibits the progression of BCa. In turn, redox changes can also influence the expression of lncRNAs^35^. The interaction between lncRNAs and redox regulation should be highlighted, as redox-related lncRNAs have been considered biomarkers for prognostic prediction in various cancers, such as lung adenocarcinoma and renal clear cell carcinoma^36,37^. Nevertheless, no data currently demonstrate whether redox-related lncRNAs could serve as useful biomarkers in BCa prognostic prediction. Hence, the role of redox-related lncRNAs in BCa requires further investigation to improve individualized therapy.

In the present study, we constructed a novel redox-related lncRNA prognostic signature for BCa and systematically investigated the associations between redox-related lncRNAs and clinical outcomes in relation to clinicopathological characteristics. In addition, we assessed the differences in cell signaling pathways, biological processes, and chemosensitivity between the low- and high-risk groups. Our findings may contribute to improved prognostic prediction and personalized treatment strategies for BCa patients.

## Results

### Identification of redox-related lncRNAs

**Fig. 1:**
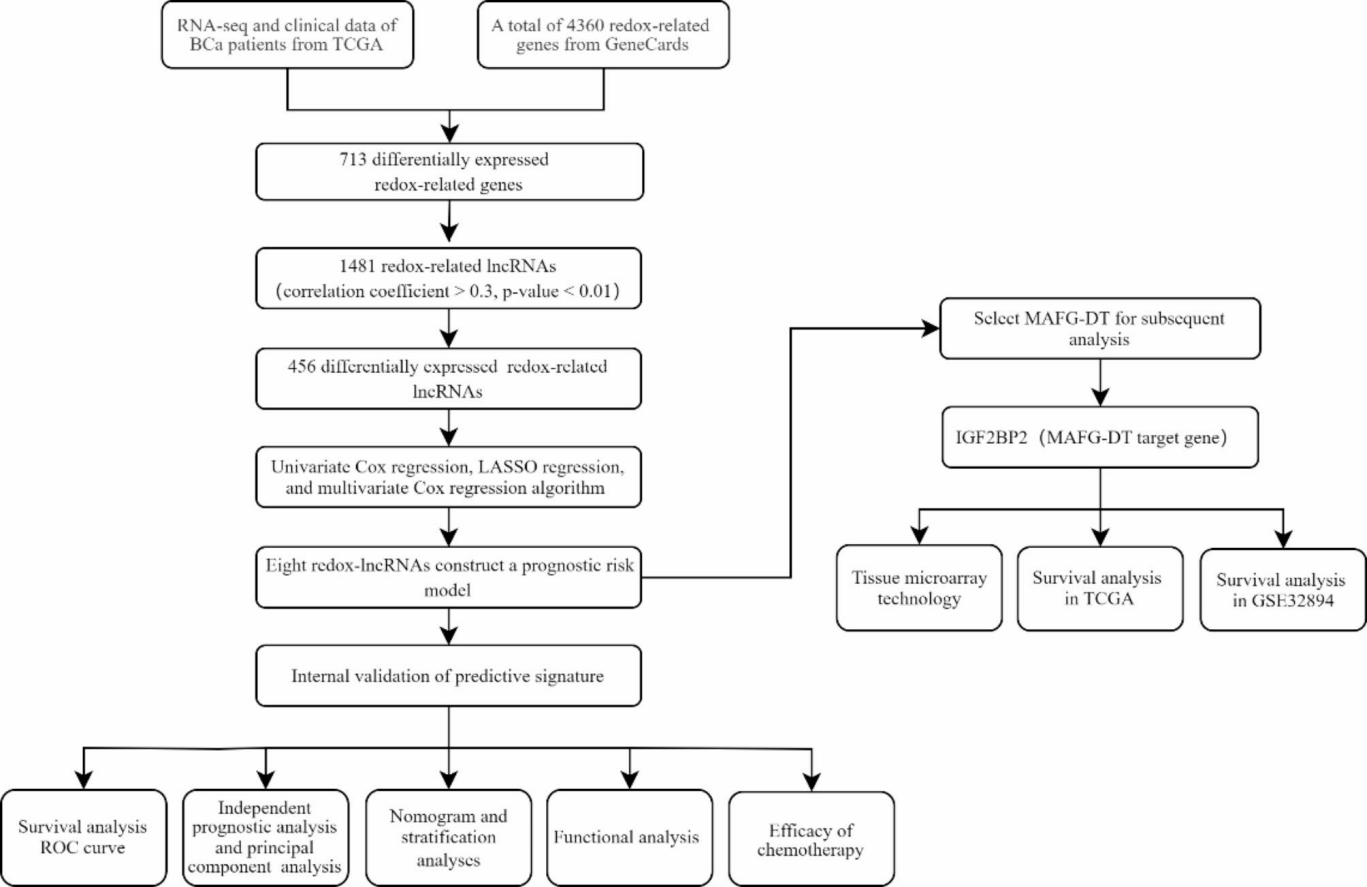
The flowchart of our study. BCa, bladder cancer; TCGA, The Cancer Genome Atlas; lncRNAs, long non-coding RNAs; LASSO regression, least absolute shrinkage and selection operator regression; ROC, receiver operating characteristic.

Figure 1 shows a flowchart of our study. A total of 713 differentially expressed redox-related genes were identified (Supplementary Fig. 1A). We identified 1,481 redox-related lncRNAs associated with differentially expressed redox-related genes and ultimately identified 456 differentially expressed redox-related lncRNAs (Supplementary Fig. 1B).

### Identification of a prognostic redox-related lncRNA signature

**Fig. 2:**
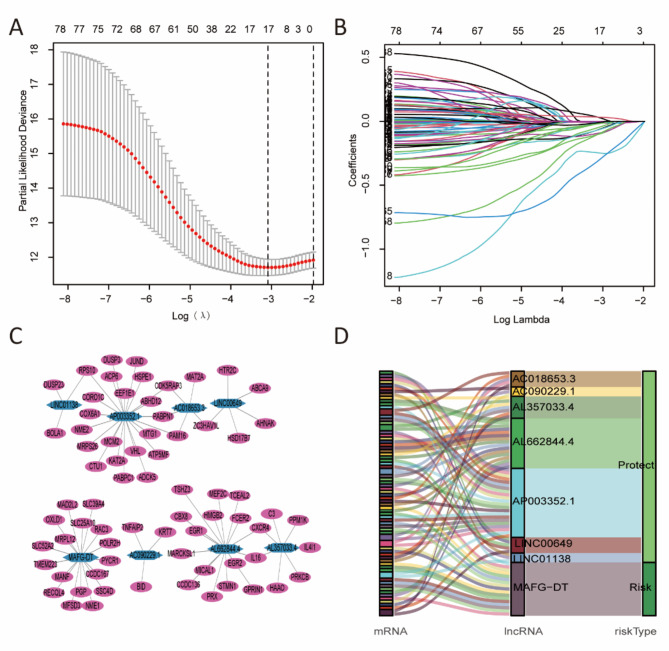
Establishment of a risk score model. (**A, B**) Based on 80 redox-related lncRNAs obtained by univariate Cox regression analysis, LASSO regression analysis identified 17 redox-related lncRNAs. (**C**) The co-expression network between lncRNAs and mRNAs. (**D**) Sankey diagram of prognostic redox-related lncRNA.

The univariate Cox regression analysis identified 80 *redox-related lncRNAs* correlated with OS. A total of 17 *redox-related lncRNAs* were screened by LASSO regression analysis (Fig. 2A, B). Multivariate Cox regression analysis identified eight lncRNAs (AC018653.3, AC090229.1, AL357033.4, AL662844.4, AP003352.1, LINC00649, LINC01138, and MAFG-DT) for the construction of prognostic model. The expression levels of the eight redox-related lncRNAs in tumor and normal tissues was shown in Supplementary Fig. 2. The risk score was calculated as follows: Risk score = (-0.4812 ×AC018653.3 expression value) + (-0.4822 × AC090229.1 expression value) + (-0.5247×AL357033.4 expression value) + (-0.8482×AL662844.4 expression value) + (-0.7467×AP003352.1 expression value) + (-0.7199×LINC00649 expression value) + (-1.2138×LINC01138 expression value) + (0.4583×MAFG-DT expression value).

We focused on the eight redox-related lncRNAs and constructed the lncRNA-mRNA co-expression network (Fig. 2C). The Sankey plot showed that MAFG-DT was the risk factor and other lncRNAs (AC018653.3, AC090229.1, AL357033.4, AL662844.4, AP003352.1, LINC00649, and LINC01138) were the protective factors (Fig. 2D).

### Evaluation of the risk model

**Fig. 3:**
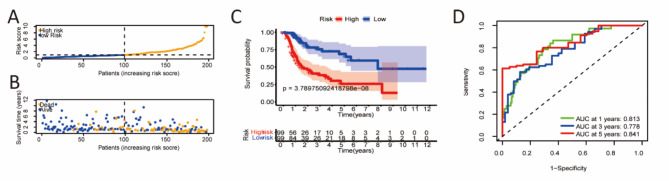
Evaluation of the risk score model. Risk scores and survival status in the first internal cohort (**A, B**). Kaplan–Meier tests in the first internal cohort (**C**). Time-dependent ROC analysis of risk score at 1, 3, and 5 years in the first internal cohort (**D**).

Patients were randomly split into two cohorts in equal ratios and assigned to low- and high-risk groups according to their median risk scores to test the predictive signature. Here, we chose the first internal cohort’s results as a representative, and the results of the second internal cohort and entire cohort were shown in the supplementary Fig. 3. Figure 3A displayed the risk scores. Figure 3B showed that survival times decreased with increasing risk scores. The OS rate in the low-risk group was significantly higher than that in the high-risk group, according to the Kaplan-Meier analysis (Fig. 3C). The time-dependent ROC curve showed area under the curve (AUC) at 1, 3, and 5 years were 0.813, 0.778, and 0.841 (Fig. 3D), respectively.

### The risk score was an independent predictor

**Fig. 4:**
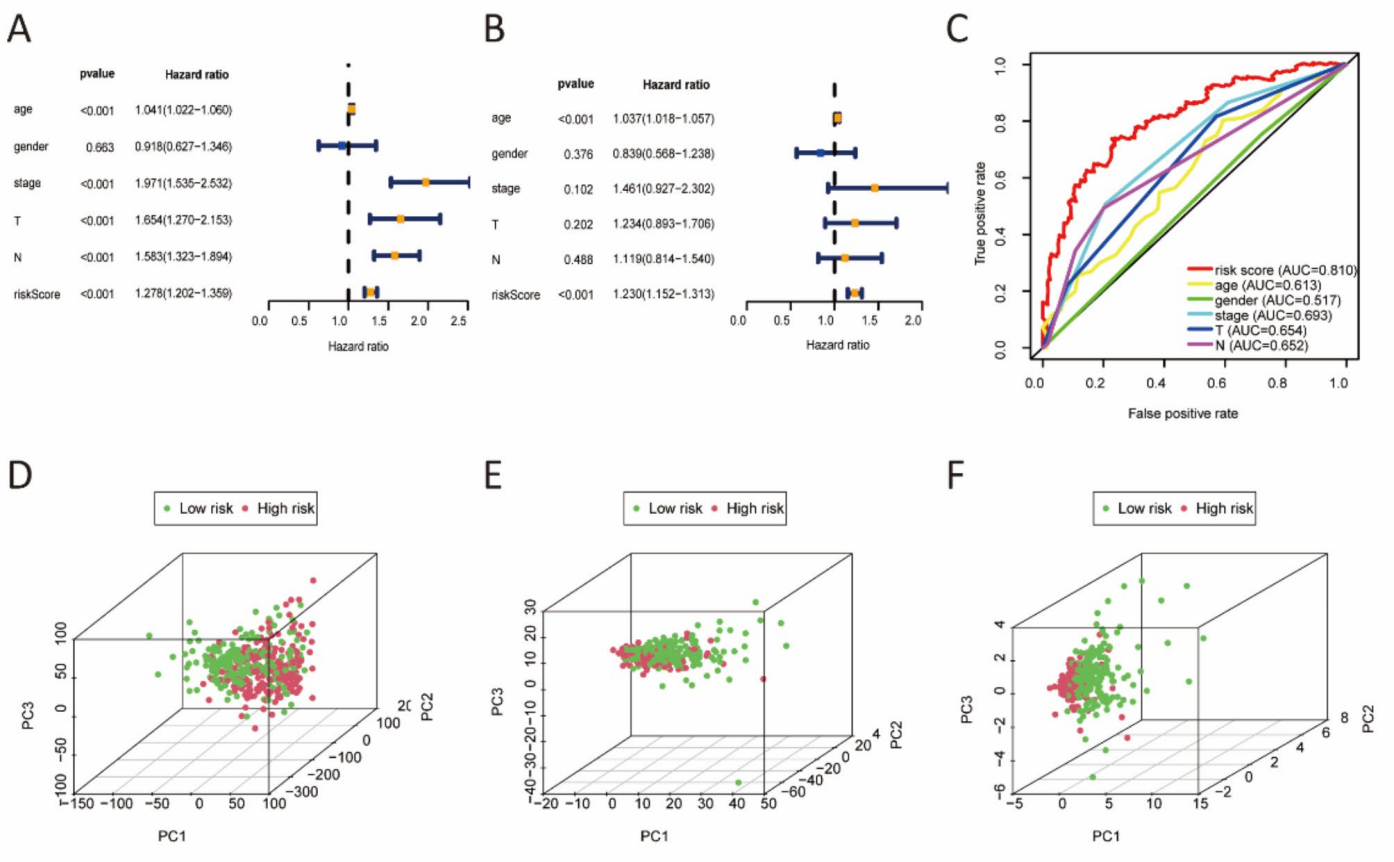
Risk score was an independent prognostic factor of OS and principal component analysis. (**A**) Univariate Cox regression analysis. (**B**) Multivariate Cox regression analysis. (**C**) Time-dependent ROC analysis for predicting OS by prognostic factors in entire cohort. (**D**) whole genes. (**E**) The 456 differentially expressed redox-related lncRNAs. (**F**) The risk model including 8 redox-related lncRNAs.

Cox regression analysis revealed that risk score and age were independent predictors for OS (Fig. 4A, B). The AUC of risk score was 0.81 in multi-index ROC analysis, which was higher than any of clinicopathological characteristic (Fig. 4C). In addition, supplementary Fig. 4 illustrated the associations between eight redox-related lncRNAs and clinical variables such as age, gender, grade, tumor stage, T, and N. Furthermore, principal component analysis (PCA) revealed that the eight redox-related lncRNAs (Fig. 4F) could discriminate patients with different risk scores better than entire genes (Fig. 4D) or 456 differentially expressed redox-related lncRNAs (Fig. 4E).

### The risk model was correlated with the prognosis in different clinicopathological features

**Fig. 5:**
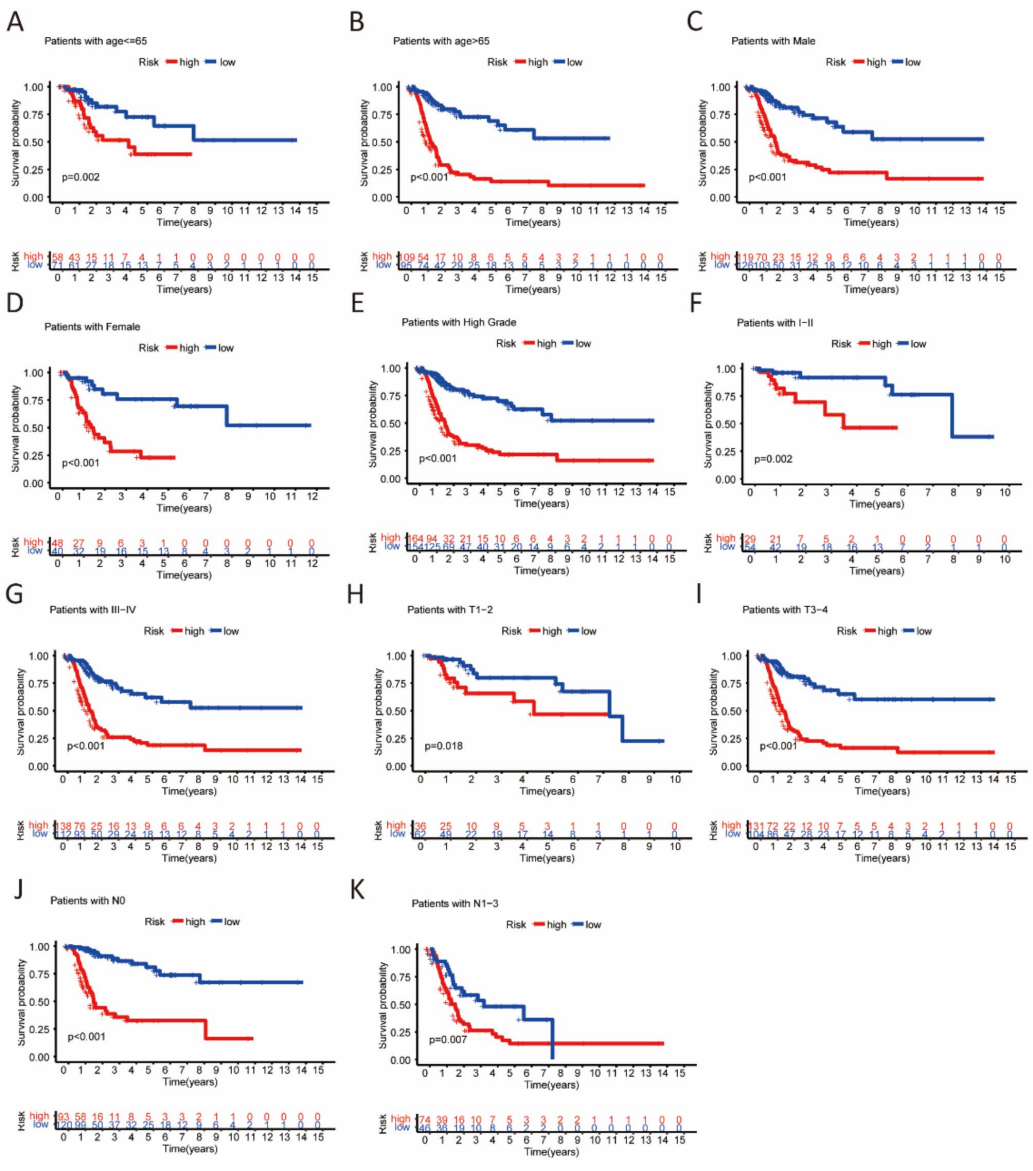
Kaplan–Meier survival analysis in different stratified clinicopathological features. (**A, B**) Age. (**C, D**) Gender. (**E**) Grade. (**F, G**) Tumor stage. (**H, I**) T stage. (**J, K**) N stage. (T represents the size and extent of the main tumor; N represents the number of nearby lymph nodes).

Patients with BCa were classified based on clinicopathologic characteristics such as age, gender, tumor stage, grade, T stage, and N stage. Survival probability was significantly higher in the low-risk group compared to the high-risk group (Fig. 5).

### Associations between redox-related lncRNAs and clinicopathological characteristics

In the prognosis risk model, eight redox-related lncRNAs were related to clinicopathological features. AC018653.3 was associated with survival status and N stage (supplementary Fig. 5A, B), while AC090229.1 was associated with survival status, grade, tumor stage, and T stage (supplementary Fig. 5C-F). AL357033.4 was associated with survival status, grade, and tumor stage (supplementary Fig. 5G-I). There was a correlation between AL662844.4 and survival status (supplementary Fig. 5J). AP003352.1 had a connection to survival status, grade, and T stage (supplementary Fig. 5K-M). LINC01138 was associated with survival status (supplementary Fig. 5N). There was an association between LINC00649 and survival status (supplementary Fig. 5O). MAFG-DT was related to survival status, age, and N stage (supplementary Fig. 5P-R).

### Nomogram construction

**Fig. 6:**
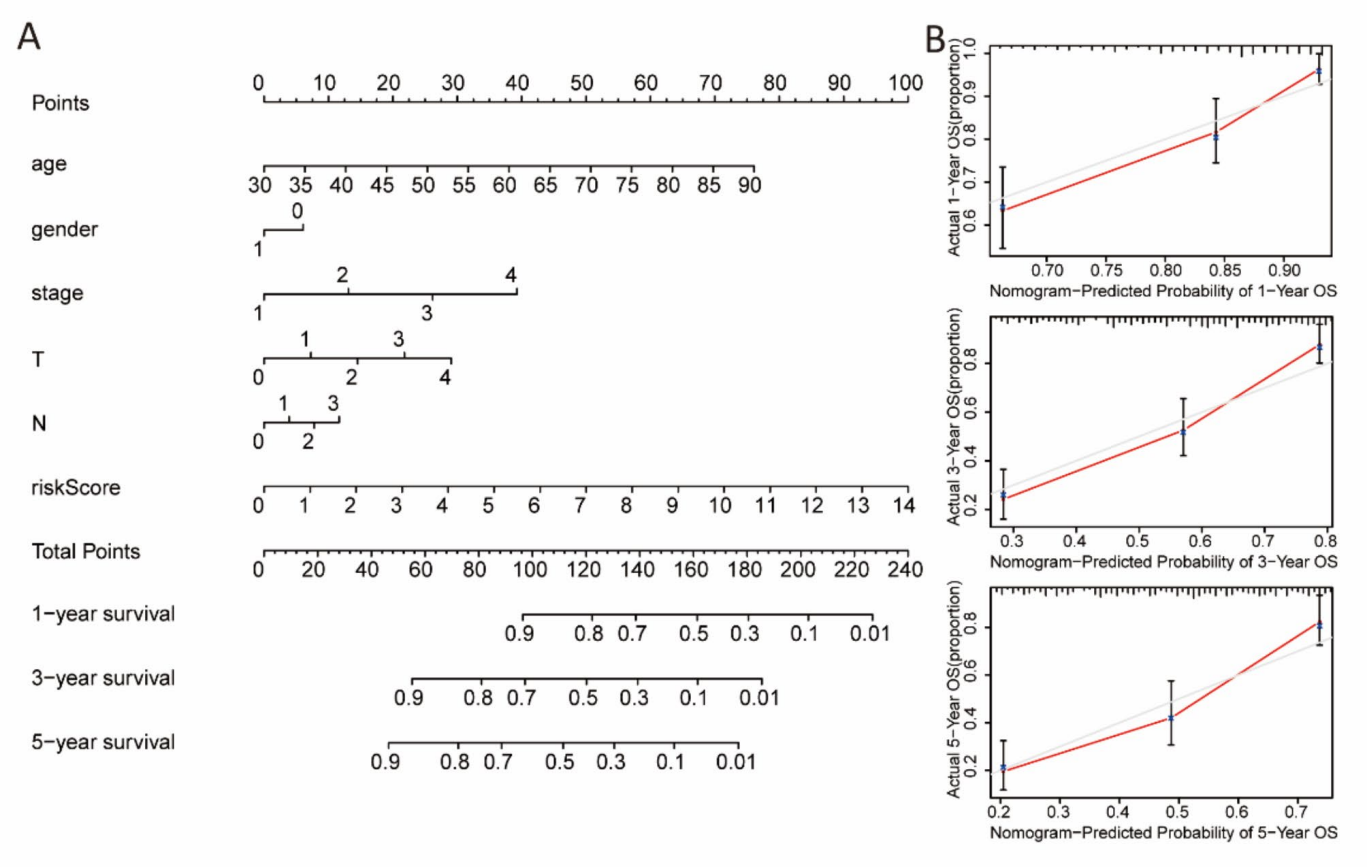
Nomogram construction and validation. (**A**) Nomogram predicting 1-year, 3-year, and 5-year prognosis based on risk score and clinicopathological features. (**B**) Nomogram-predicted probability of 1-year, 3-year, and 5-year survival.

The nomogram, which included the risk score and clinicopathological characteristics, was able to predict the 1-year, 3-year, and 5-year prognosis of BCa patients (Fig. 6A). The predicted survival rates corresponded closely with the actual OS rates at 1-year, 3-year, and 5-year intervals, confirming the nomogram’s strong predictive capacity (Fig. 6B).

### Gene set enrichment analysis

**Fig. 7:**
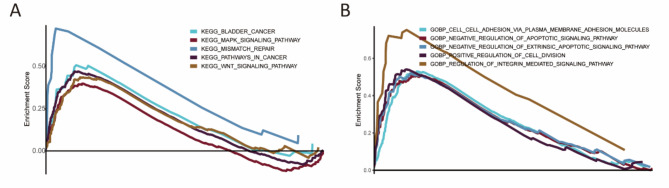
Gene Set Enrichment Analysis. (**A**) KEGG pathway analysis showed five pathways were enriched in high-risk group. (**B**) Five GO items were enriched in the high-risk group.

GSEA showed that KEGG pathways such as BCa, the MAPK signaling pathway, the Wnt signaling pathway, mismatch repair, and pathways in cancer were significantly enriched in the high-risk group, while no pathways were enriched in the low-risk group (Fig. 7A). In terms of GO analysis, the high-risk group enriched cell adhesion via plasma membrane adhesion molecules, negative regulation of apoptotic signaling pathway, negative regulation of extrinsic apoptotic signaling pathway, positive regulation of cell division, and regulation of integrin mediated signaling pathway, while the low-risk group did not show enrichment in any items (Fig. 7B).

### Associations between the risk model and chemosensitivity

**Fig. 8:**
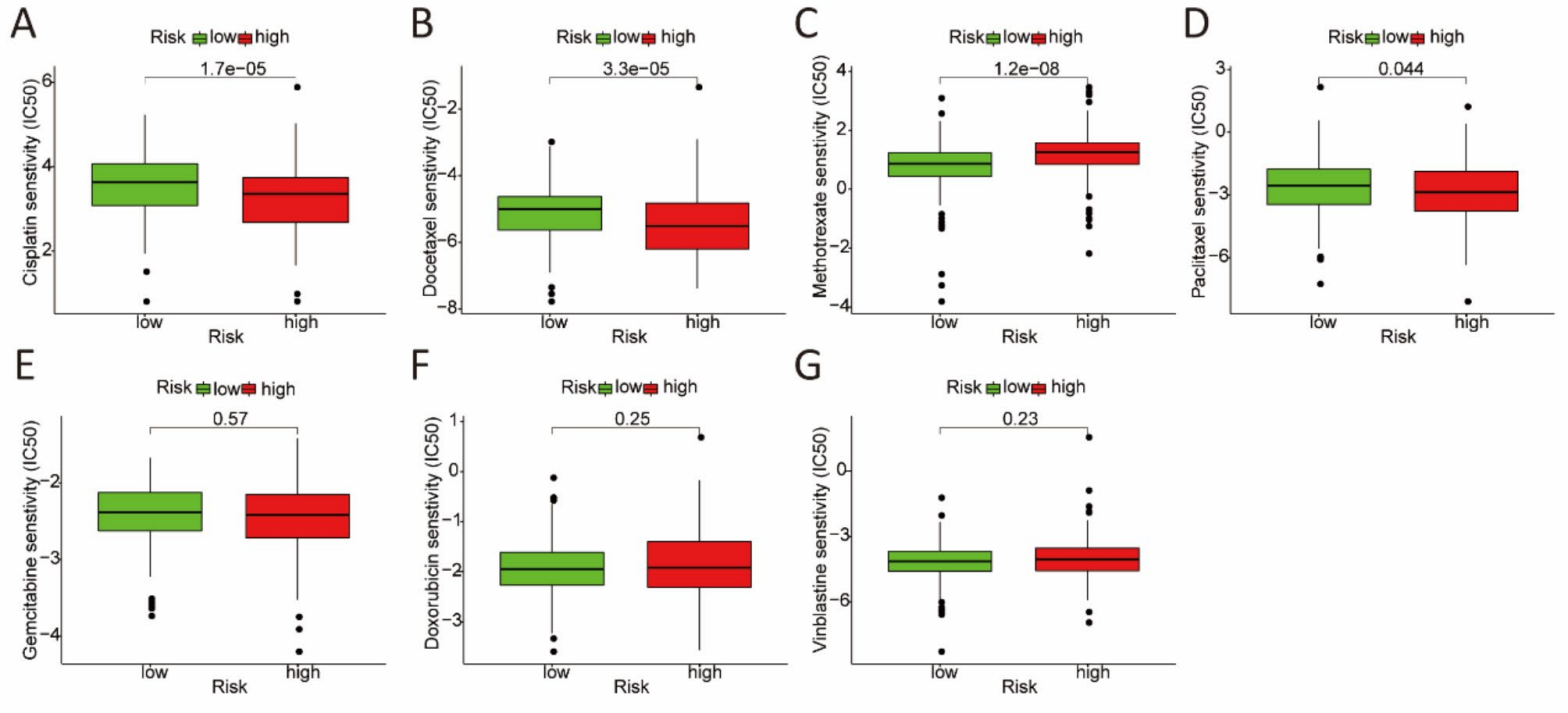
The efficacy of BCa treatment in the low-risk and high-risk groups. The IC50 of (**A**) Cisplatin, (**B**) Docetaxel, (**C**) Methotrexate, (**D**) Paclitaxel, (**E**) Gemcitabine, (**F**) doxorubicin, and (**G**) Vinblastine was compared between low-risk and high-risk groups.

The association between the risk model and the efficacy of general chemotherapy in BCa patients was investigated. The results revealed that the IC50 values of cisplatin, docetaxel, and paclitaxel were higher in the low-risk group, whereas methotrexate had a higher IC50 value in the high-risk group (Fig. 8A-D). In addition, there was no significant difference in the IC50 values of gemcitabine, doxorubicin, and vinblastine between the low- and high-risk groups (Fig. 8E-G).

### IGF2BP2 was highly expressed in tumor tissues and correlated with overall survival

**Fig. 9:**
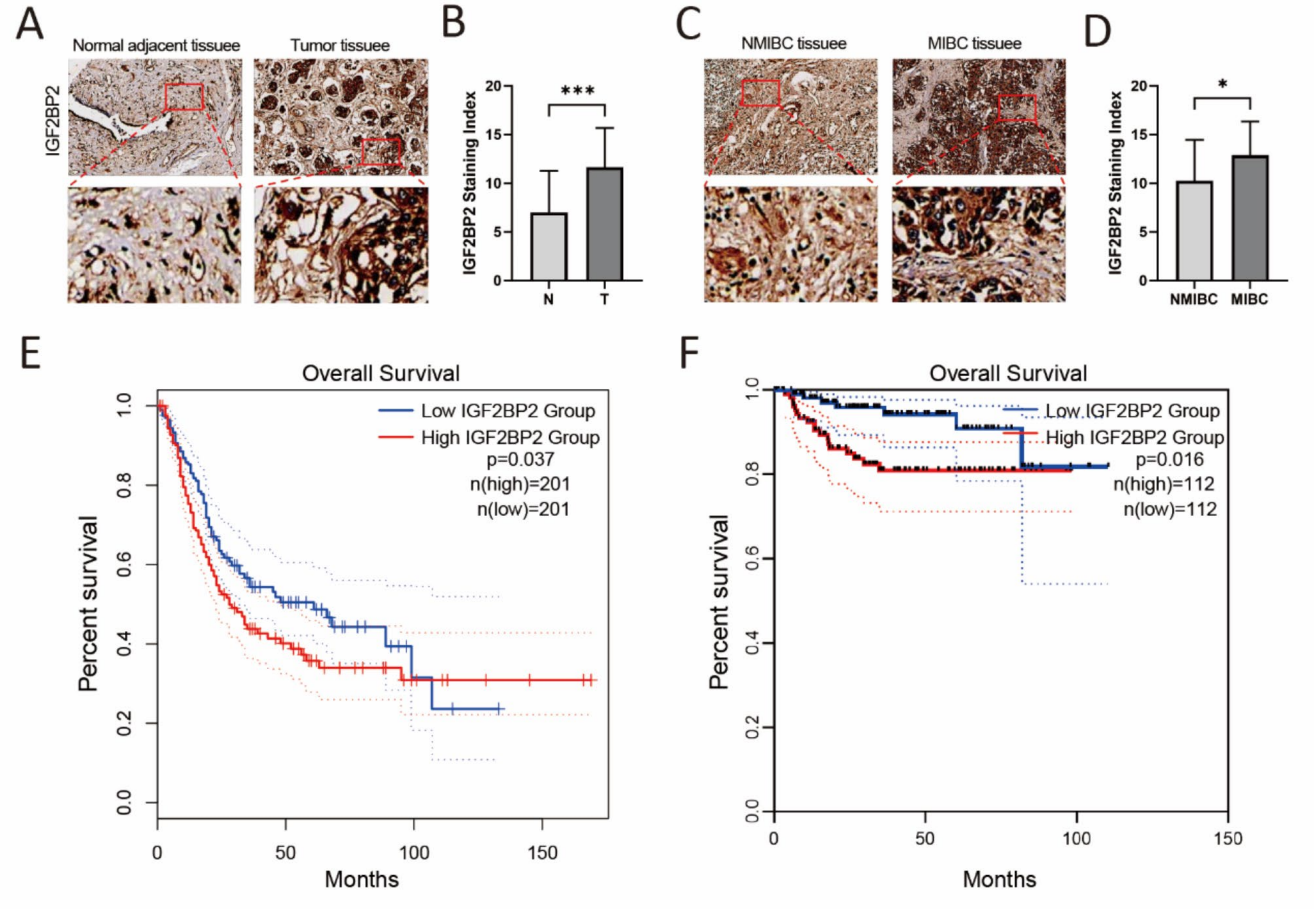
The expression of IGF2BP2 was analyzed by tissue microarray study and overall survival analysis. (**A** and**C**) Representative images of IGF2BP2 immunohistochemistry in BCa tissues and normal adjacent bladder tissues. (**B** and**D**) Staining index of IGF2BP2 in different groups. (**E**) Survival analysis of IGF2BP2 in BCa patients in the TCGA database (Figure E was obtained from GEPIA 2, http://gepia2.cancer-pku.cn/). (**F**) Survival analysis of IGF2BP2 in GSE32894. (*p < 0.05,***p < 0.001; MIBC represents muscle-invasive bladder cancer and NMIBC represents non-muscle-invasive bladder cancer).

Since MAFG-DT was the only identified risk factor, IGF2BP2, as one of the target genes of MAFG-DT, was selected for the following studies. The tumor tissues stained more strongly with anti-IGF2BP2 antibody than normal adjacent bladder tissues (Fig. 9A). The staining index of IGF2BP2 was significantly higher in tumor tissues (Fig. 9B). Additionally, non-muscle-invasive bladder cancer (NMIBC) tissues stained poorly with anti-IGF2BP2 antibody compared to muscle-invasive bladder cancer (MIBC) tissues (Fig. 9C). The IGF2BP2 staining index was significantly higher in the MIBC group compared to the NMIBC group (Fig. 9D). Figure 9E was obtained from the analysis of the TCGA database using GEPIA 2 (http://gepia2.cancer-pku.cn/), and Fig. 9F was obtained by performing survival analysis on GSE32894. IGF2BP2 expression was significantly and negatively correlated with the OS of BCa patients in both databases (Fig. 9E, F).

## Discussion

Both redox alteration and lncRNAs are both responsible for the development and progression of BCa, and redox-related lncRNAs have been utilized as useful prognostic biomarkers in several cancers. However, limited information is available regarding the role of redox-related lncRNAs in predicting BCa prognosis.

Based on public database (TCGA and GeneCards), we constructed a redox-related lncRNA risk model for BCa prognosis prediction. In the present study, eight redox-related lncRNAs (AC018653.3, AC090229.1, AL357033.4, AL662844.4, AP003352.1, LINC00649, LINC01138, and MAFG-DT) with prognostic significance were ultimately identified using LASSO regression analysis and Cox regression analysis. Among the identified lncRNAs, MAFG-DT (MAFG-AS1) is the only hazardous factor, which is involved in BCa progression. Previous studies have reported that MAFG-DT is associated with a variety of tumors, including BCa^38^, liver cancer^39^, and breast cancer^40^. Additionally, MAFG-DT also regulates several tumor-related pathways, such as HuR/PTBP1 axis^38^, miR-143-3p/SERPINE1 axis^41^, and miR-125b-5p/SphK1 axis in BCa^42^. With regard to the other redox-related LncRNAs in our risk model, they are all protective factors. AC018653.3 and AP003352.1 are ferroptosis-related^43^, while AL357033.4 is immune-related and links to muscle-invasive BCa^44^. Similarly, AL662844.4 has been used in an autophagy-related lncRNA prognostic model of BCa^45^, and LINC01138 together with other lncRNAs has been proven to comprise a signature for predicting the survival of patients with BCa^46^. Several studies indicated that LINC00649 is a protective factor which is consistent with our analysis^44,47^. But for AC090229.1, unfortunately, no data are currently available in cancer research. It is postulated that the AC090229.1 might be a unique factor associated with redox regulation in BCa. Overall, these results indicated that these lncRNAs in our risk model are promising prognostic indicators of BCa.

The risk score for each patient was determined by the formula and all patients in different groups were split into high-risk and low-risk subgroups according to the median value. Compared to the low-risk patients, the high-risk patients had shorter OS time, as confirmed by time-dependent ROC curve. Internal verification demonstrated that the predictive signature performed effectively in prognostic prediction. Furthermore, the OS of BCa patients was closely associated with age, tumor stage, T stage, and N stage, and the risk score had greater accuracy in predicting the prognosis compared to clinicopathological variables, as verified by the nomogram.

Concerning the signaling pathway, the MAPK and Wnt pathways have been found enriched in the high-risk group according to the GSEA results. The MAPK signaling pathway is closely related to cancer progression and metastasis. HnRNP-L strengthens BCa progression through increasing the MAPK signaling pathway and inhibiting intrinsic apoptotic signaling^48^. TRPM7 modulates MAPK signaling pathway and affects the activities and apoptosis of BCa cells^49^. BCa progression and invasion are regulated by galectin-1 via the MAPK signaling pathway^50^. RAB14 mediates the invasion and metastasis of BCa cells via activating MAPK signaling pathway^51^. The Wnt signaling pathway is also tightly associated with cancer. Silencing lncRNA GTF2IRD2P1 promotes BCa progression by targeting the Wnt signaling pathway^52^. ZEB1-mediated biogenesis of circular RNA NIPBL sustains BCa metastasis by Wnt signaling pathway^53^. Decreased expression of lncRNA CASC2 facilitates cell proliferation and metastasis of BCa via activation of the Wnt signaling pathway^54^. Altogether, this indicates that the two pathways may contribute the poor prognosis of patients in the high-risk group. In addition, negative regulation of apoptotic signaling pathway and positive regulation of cell division were enriched in the high-risk group, which may partially explain why the patients in the high-risk group had a poor prognosis. Furthermore, we also investigated the chemosensitivity between the two groups and showed that cisplatin, docetaxel, and paclitaxel were more sensitive to the patients in the high-risk group, whereas methotrexate was more sensitive to the patients in the low-risk group. The results may provide individualized chemotherapy for patients with BCa.

To date, multiple signatures have been established and validated for predicting the prognosis of BCa patients. Genes related to the autophagy-immune crosstalk, the ferroptosis process, and the m6A-immnue network have been reported as BCa prognostic markers^55–58^. Our redox-lncRNA signature shows higher predictive accuracy, as indicated by a higher AUC, in BCa prognosis compared to the majority of prognostic signatures.

As a predicted target gene of MAFG-DT, IGF2BP2 was highly expressed in tumor tissues in our study, especially in high pathological stages. And IGF2BP2 were associated with the OS of BCa patients according to the survival analysis in our research. Previous studies have found that IGF2BP2 overexpression occurs in a variety of cancers and is associated with tumor growth and migration^59–61^. In addition, expression of IGF2BP2 was found to be upregulated in BCa cell lines and tissues compared to urothelial cell lines and adjacent normal tissues, respectively^62^, which is consistent with our findings. Hence, IGF2BP2 might be considered a promising biomarker for BCa diagnosis and targeted therapy.

Despite the contributions of our study, some limitations remain. First, the redox-lncRNA predictive signature was established by bioinformatics analysis, and further molecular experiments are required to confirm our results. Second, the mechanisms underlying the lncRNA-based signaling mediated by redox in BCa were not fully clarified. Third, the sample size for immunohistochemistry was limited, and expansion of the cohort is necessary. Additionally, comprehensive follow-up information, including long-term survival rate and drug sensitivity, is needed to strengthen our conclusions.

## Materials and methods

### Data acquirement and clinical samples

RNA-sequencing data of BCa (*n* = 412) and normal tissue samples (*n* = 19), along with clinical data of BCa patients, were obtained from the TCGA database (https://portal.gdc.cancer.gov/). After removing samples missing clinical features or overall survival (OS) of less than 30 days, 400 BCa samples were retained, and the complete clinical data of BCa patients are shown in Supplementary Table 1. The GSE32894 dataset was retrieved from the Gene Expression Omnibus (GEO) (https://www.ncbi.nlm.nih.gov/geo/) and used for survival analysis. Additionally, two paraffin-embedded tissue microarrays containing 60 BCa samples and 42 adjacent normal bladder samples were obtained from the Third Medical Center, Chinese People’s Liberation Army (PLA) General Hospital, between 2021 and 2023. Ethics review was approved by the Medical Ethics Committee of the Chinese People’s Liberation Army (PLA) General Hospital (NO.22QNFC095), and informed consent was obtained from all participants in accordance with institutional guidelines.

### Identification of redox-related lncRNAs

In total, 4360 redox-related genes were collected from GeneCards (https://www.genecards.org/). We obtained differentially expressed genes through limma R package (thresholds of |log fold change (FC)| > 1 and false discovery rate (FDR) < 0.05) (version 3.53.10). Using the screening criteria (|correlation coefficient|> 0.3, p-value < 0.01), we identified correlations between redox-related genes and lncRNAs using limma package.

### Redox-related lncRNA prognostic model construction

We obtained redox-related lncRNAs associated with the prognosis of BCa patients by univariate Cox regression (*p* < 0.05). Subsequently, least absolute shrinkage and selection operator (LASSO) regression analysis and multivariate Cox regression analysis were conducted to select suitable redox-related lncRNAs for constructing this model (*p* < 0.05). The risk score was determined by the following formula: risk score = Ʃ[Coef(lncRNA) × Exp(lncRNA)], where Coef represents the coefficient value and Exp represents the expression value of the lncRNA. We used the “survival,” “glmnet,” “survminer,” “caret,” “pheatmap,” “ggplot2,” “ggalluvial,” “dplyr,” and “survivalROC” R packages for these analyses. The network between lncRNAs and mRNAs was visualized using Cystoscape software.

### Redox-related lncRNA prognostic risk model verification

Time-dependent receiver operator characteristic (ROC) analysis, risk score analysis, Kaplan–Meier analysis, and survival outcome analysis were conducted to identify prognostic differences between the low- and high-risk groups. We used univariate and multivariate Cox regression analysis and multi-index ROC analysis to determine whether this model served as an independent prognostic predictor of OS. Additionally, the relationship between lncRNAs/risk score and clinical characteristics was evaluated. We performed the “survivalROC” “survminer,” “pheatmap,” “timeROC,” “beeswarm,” and “survival” R packages for analysis.

### Nomogram establishment

Risk score and clinicopathological factors including age, gender, stage, T, and N were utilized to construct a nomogram capable of predicting the 1-, 3-, and 5-years survival of BCa patients. We used calibration curve to predict the accuracy of established nomogram. The “rms” R package was conducted to analysis.

### Gene set enrichment analysis (GSEA)

The biological functions between the low- and high-risk groups were explored by GSEA software (version 4.2.1) (Nominal p value < 0.05, FDR < 0.05). Kyoto Encyclopedia of Genes and Genomes (KEGG) enrichment analysis was utilized to evaluated signaling pathways. Gene ontology (GO) analysis was performed to evaluate biological processes and molecular function.

### Clinical treatment response prediction

The half-maximal inhibitory concentration (IC50) of selected drug candidates was calculated for different groups using the “pRRophetic” and “ggplot2” packages (*P* < 0.05).

### Prediction of lncRNA target genes and tissue microarray technology

The lncRNA target genes were predicted using catRAPID omics (http://service.tartaglialab.com/). After being deparaffinized in environmental dewaxing dip wax transparentize solution, the paraffin-embedded tissue microarrays were rehydrated in a graded ethanol series. Antigen retrieval involved heating the slides in 0.01 M citrate-buffered solution (pH 6.0) using an electric constant temperature drying oven (60 ℃, 30 min). Endogenous peroxidase activity was quenched with 3% hydrogen peroxide diluted in double-distilled water for 25 min. Subsequently, the slides were incubated overnight at 4 ℃ with primary antibody against QKI rabbit monoclonal antibody (dilution 1:200, AB126742, Abcam) or IGF2BP2 rabbit polyclonal antibody (dilution 1:200, 11601-1-AP, PTGLAB), following incubation with 5% bovine serum albumin. HRP-conjugated goat anti-rabbit IgG (dilution 1:200, GB23303, Servicebio) served as secondary antibody. Color development was performed by applying 3,3’-diaminobenzidine (DAB) tetrahydrochloride, followed by counterstaining the slides with hematoxylin. A semiquantitative approach was employed for immunohistochemistry analysis^63^. In brief, the percentage of tumor cells that stained positively was defined as follows: 0 (no positive), 1 (0–10%), 2 (10–30%), 3 (30–70%), and 4 (over 70%). Staining intensity was assigned as follows: 1 (no staining), 2 (weak staining), 3 (moderate staining), and 4 (strong staining). The staining index (SI) was calculated using the formula = percentage value × staining intensity value. The cutoff value was set at the median value of SI = 8. Consequently, samples with SI ≥ 8 were classified as having high expression, while those with SI < 8 were classified as having low expression.

## Statistical analysis

All analyses were conducted in R software (version 4.2.1), except for the section “clinical treatment response prediction” (version 3.6.3). The Wilcoxon test was utilized to compare gene expression levels between tumor and control groups. OS between low- and high-risk groups was evaluated by Kaplan-Meier survival curves and log-rank analysis. The staining index between BCa tissues and normal adjacent bladder tissues was compared using a t-test. Differences were deemed to be statistically significant if *p* < 0.05.

## Conclusions

A redox-related lncRNA signature was successfully developed and verified for predicting the overall survival of BCa patients. The signature is independent of other clinical variables and more accurately predicts the prognosis of BCa patients. Our findings provide new individualized treatment guidance for BCa patients requiring chemotherapy and present potential biomarkers for BCa prognosis. It will make treatment decisions easier and more accurate for clinicians in the future. In addition, our findings demonstrate that IGF2BP2 may be a promising target for the diagnosis and treatment of BCa.

## Electronic supplementary material

Below is the link to the electronic supplementary material.

Supplementary Material 1

## Data Availability

The datasets generated during the current study are available in the in TCGA database (https://portal.gdc.cancer.gov/) and Gene Expression Omnibus (GEO) (https://www.ncbi.nlm.nih.gov/geo/).
