# Supplementary material for: A redox-related lncRNA signature in bladder cancer
Source: Sci Rep. 2024 Nov 16;14:28323. doi: 10.1038/s41598-024-80026-9 (PMC11569154; doi:10.1038/s41598-024-80026-9)
Supplement: Supplementary file 1 — Supplementary Material 1 [file 41598_2024_80026_MOESM1_ESM.docx]

**A redox-related lncRNA signature in bladder cancer**

Fuguang Zhao^1,2, †^, Hui Xie^3, †^, Yawei Guan^1,2^, Jingfei Teng^1,2^, Zhihui Li^2^, Feng Gao^2^, Xiao Luo^2^, Chong Ma^1,2,^ * and Xing Ai^1,2,^ *

^1^ Department of Urology, The Third Medical Center, Chinese People’s Liberation Army (PLA) General Hospital, Beijing 100039, P.R. China

^2^ Department of Urology, The Seventh Medical Center, Chinese People’s Liberation Army (PLA) General Hospital, Beijing 100700, P.R. China

^3^ Department of Urology, The First Affiliated Hospital of Fujian Medical University, Fuzhou 350005, P.R. China

* Corresponding authors: Chong Ma, machong314@163.com; Xing Ai, aixing0007@163.com

^†^ Contributed equally.

| **Characteristic** | **Variable** | **Total (n = 412)** | **%** |
| --- | --- | --- | --- |
| Age(years) | ≤ 65 | 162 | 39.32 |
|  | > 65 | 250 | 60.68 |
| Gender | Male | 304 | 73.79 |
|  | Female | 108 | 26.21 |
| Grade | High grade | 388 | 94.17 |
|  | Low grade | 21 | 5.10 |
|  | Unknown | 3 | 0.73 |
| Stage | I | 2 | 0.49 |
|  | II | 131 | 31.80 |
|  | III | 141 | 34.22 |
|  | IV | 136 | 33.01 |
|  | Unknown | 2 | 0.49 |
| Tumor classification | T0 | 1 | 0.24 |
|  | T1 | 3 | 0.73 |
|  | T2 | 120 | 29.13 |
|  | T3 | 196 | 47.57 |
|  | T4 | 59 | 14.32 |
|  | TX | 1 | 0.24 |
|  | Unknown | 32 | 7.77 |
| Lymph nodes | N0 | 239 | 58.01 |
|  | N1 | 47 | 11.41 |
|  | N2 | 76 | 18.45 |
|  | N3 | 8 | 1.94 |
|  | NX | 36 | 8.74 |
|  | Unknown | 6 | 1.46 |
| Distant metastasis | M0 | 196 | 47.57 |
|  | M1 | 11 | 2.67 |
|  | MX | 202 | 49.03 |
|  | Unknown | 3 | 0.73 |
| Survival status | Alive | 253 | 61.41 |
|  | Death | 159 | 38.59 |

*Supplementary Table 1. Complete clinical data of bladder cancer patients from the TCGA database. (T0 means main tumor cannot be found; T1, T2, T3, and T4 mean the size and/or extent of the main tumor; TX means main tumor cannot be measured. N0 means no cancer in nearby lymph nodes; N1, N2, and N3 mean the number and location of lymph nodes that contain cancer; NX means cancer in nearby lymph nodes cannot be measured. M0 means cancer has not spread to other parts of the body; M1 means cancer has spread to other parts of the body; MX means metastasis cannot be measured.)*


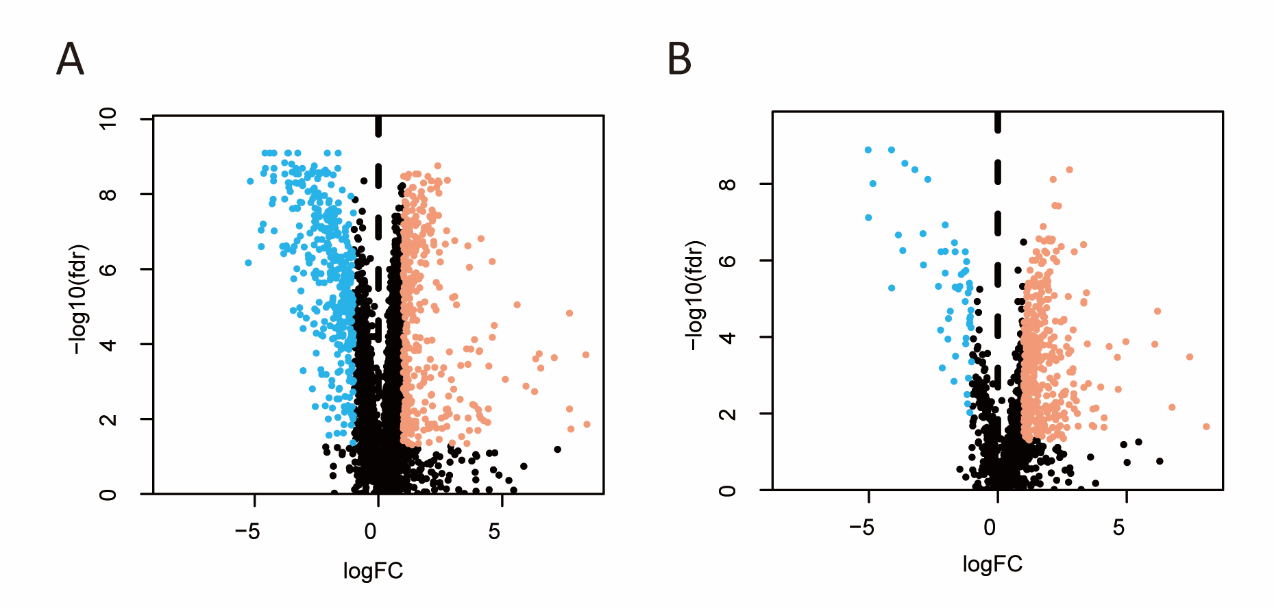


*Supplementary Figure 1. Identification of differentially expressed redox-related genes in BCa. (A) Volcano plot of redox-related genes. Blue dots: down-regulation. Orange dots: up-regulation. (B) Volcano plot of redox-related lncRNAs. Blue dots: down-regulation. Orange dots: up-regulation.*


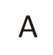
*
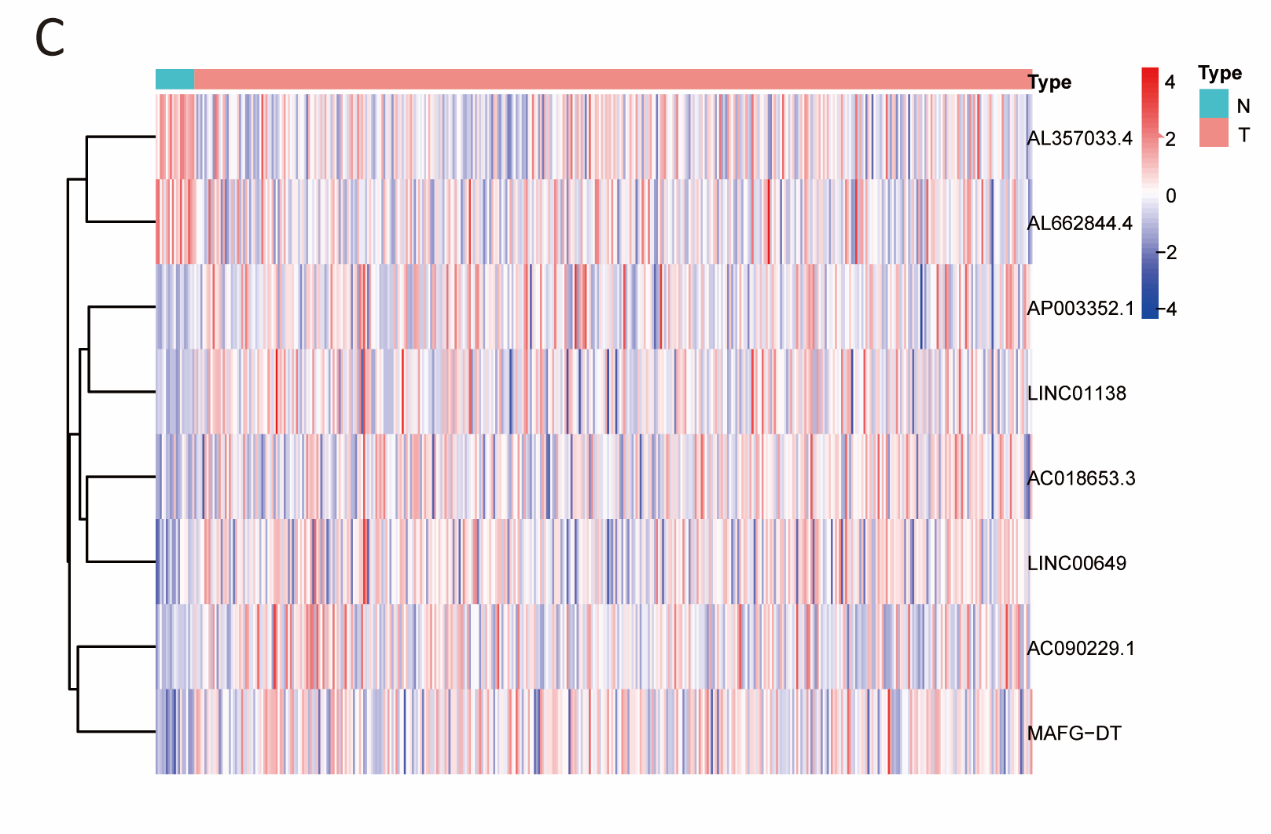
*

*Supplementary Figure 2. The expression value of eight redox-related lncRNAs in tumor and normal tissues (N: normal, T: tumor).*


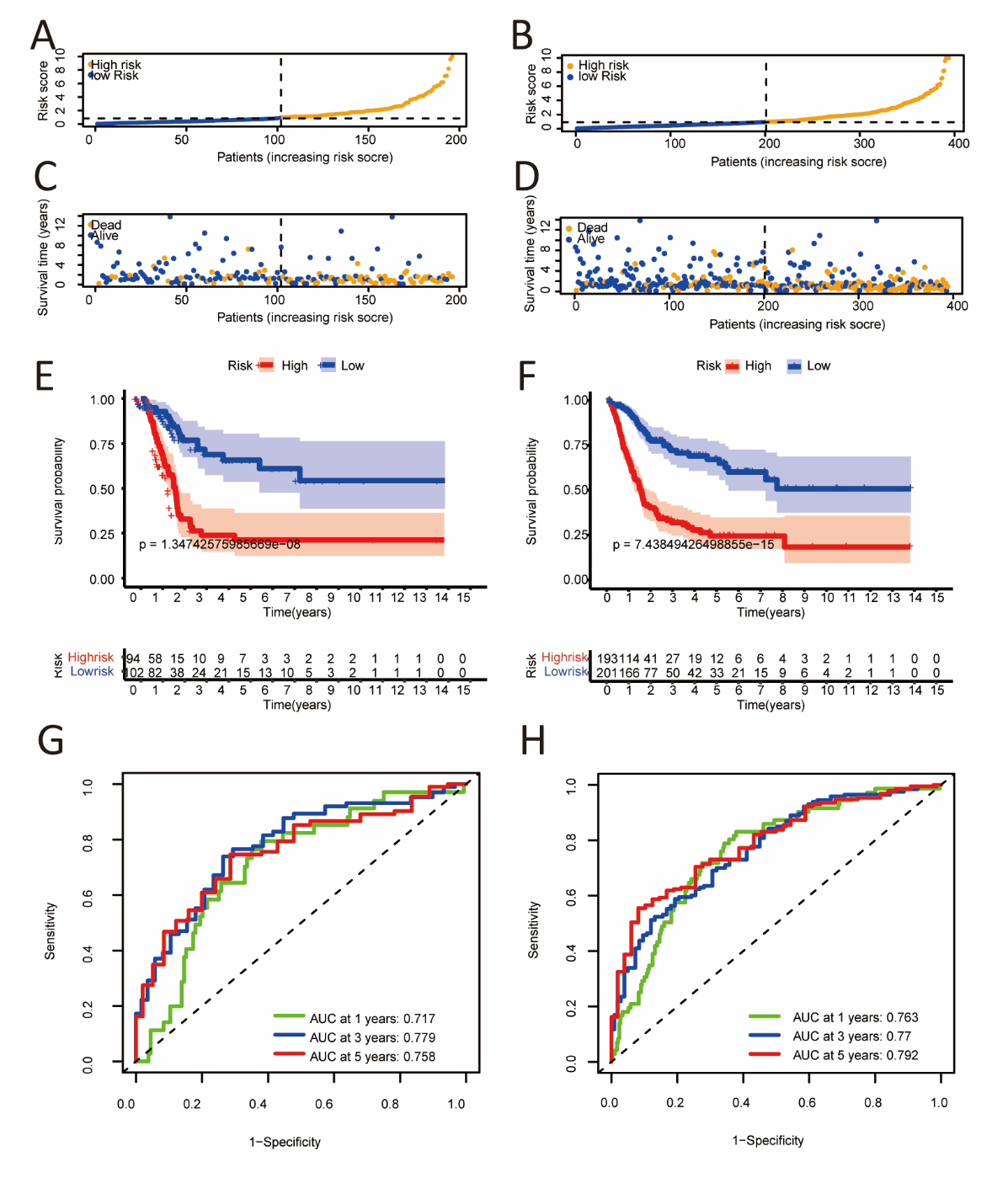


*Supplementary Figure 3. Evaluation of the risk score model. Risk scores and survival status in second internal cohort (A, C) and entire cohort (B, D). Kaplan–Meier tests in second internal cohort (E) and entire cohort (F). Time-dependent ROC analysis of risk score at 1, 3, and 5 years in second internal cohort (G) and entire cohort (H).*


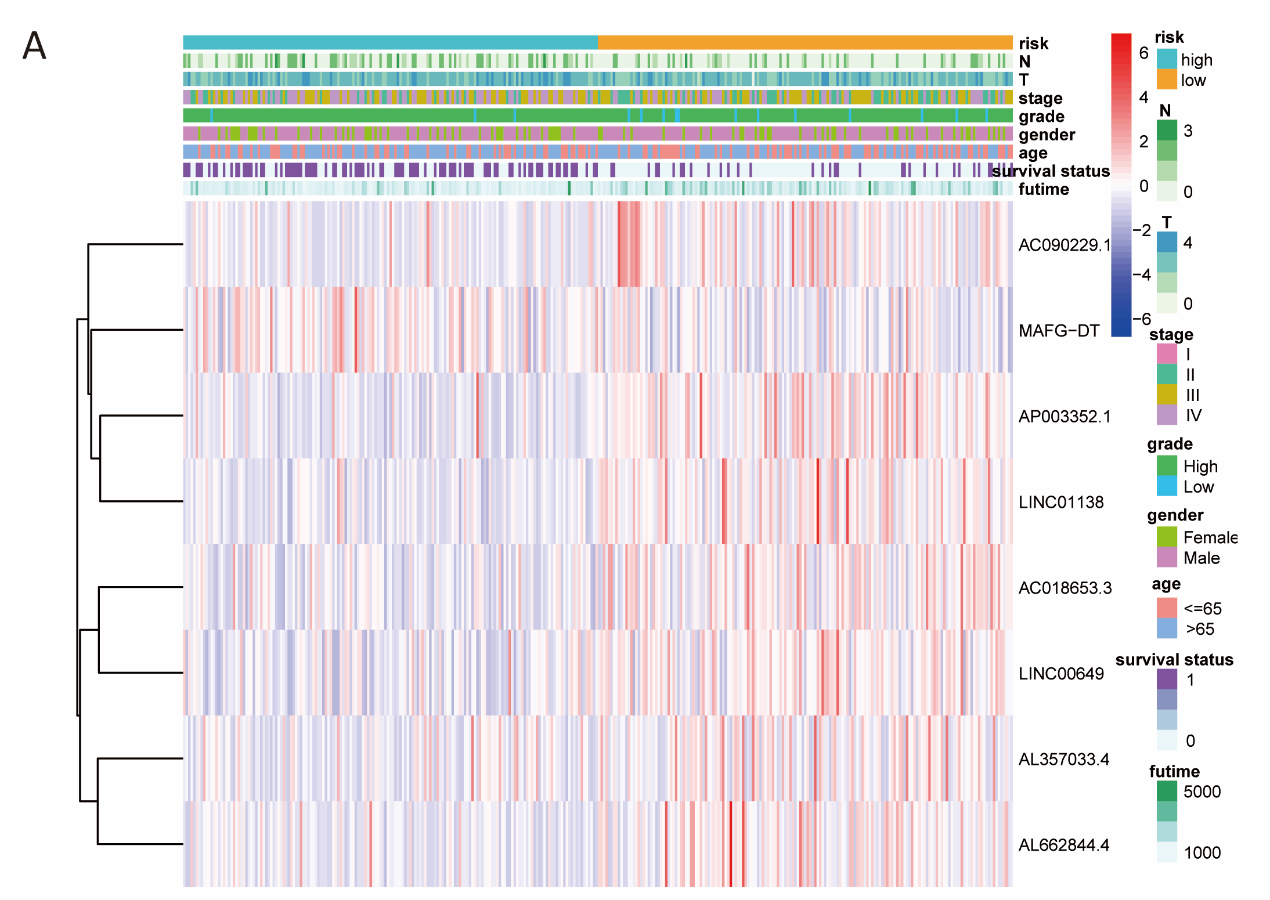


*Supplementary Figure 4. Heat map shows the correlation between the 8 redox-related lncRNAs and clinical traits in the low- and high-risk groups in entire cohort (T represents the size and extent of the main tumor; N represents the number of nearby lymph nodes).*


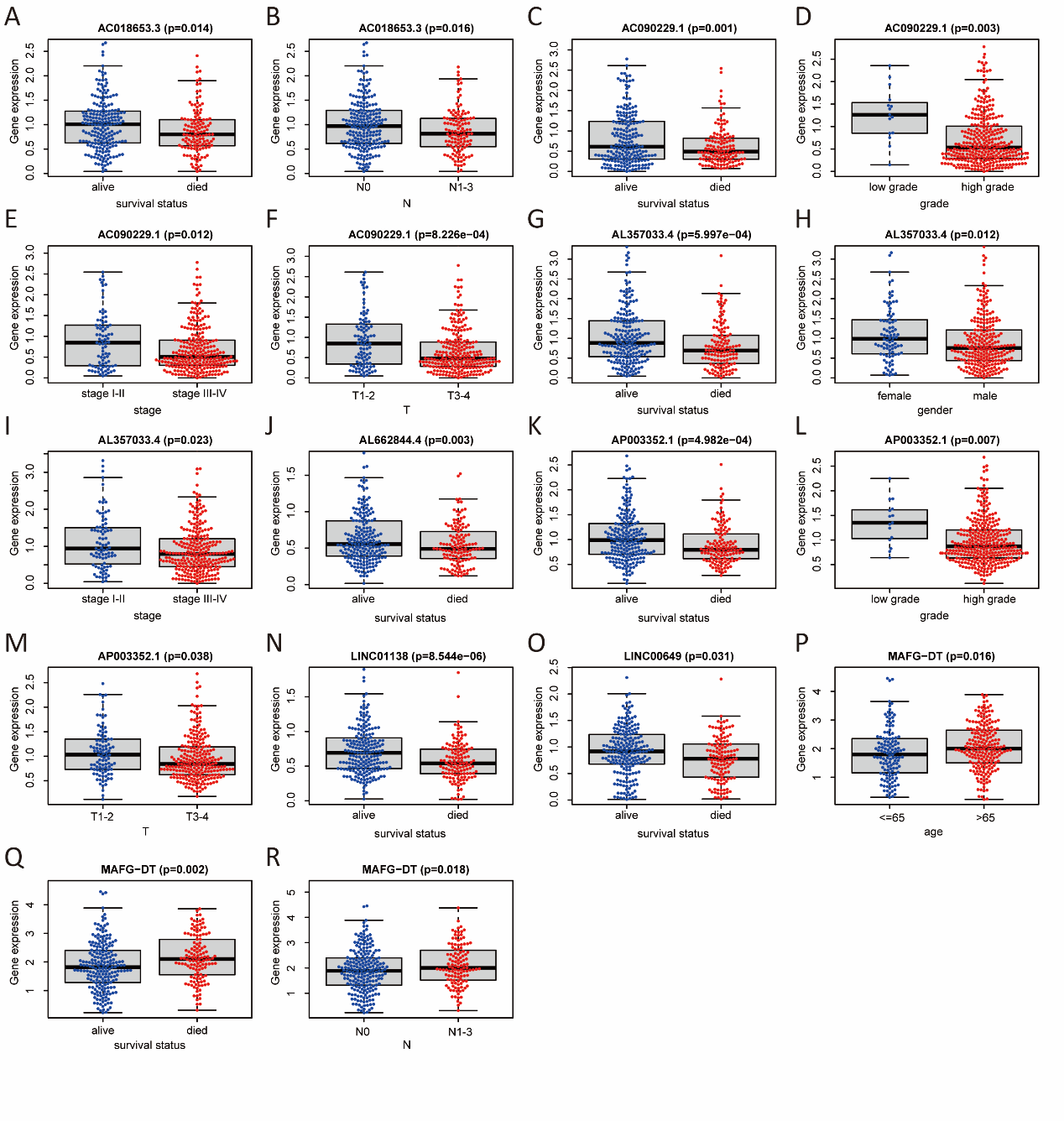


*Supplementary Figure 5. Relationships between the 8 redox-related lncRNAs and clinicopathological features. (A, B) AC018653.3, (C-F) AC090229.1, (G-I) AL357033.4, (J) AL662844.4, (K-M) AP003352.1, (N) LINC01138, (O) LINC00649, and (P-R) MAFG-DT (T represents the size and extent of the main tumor; N represents the number of nearby lymph nodes).*
